# VO_2_/TiO_2_ Nanosponges as Binder-Free Electrodes for High-Performance Supercapacitors

**DOI:** 10.1038/srep16012

**Published:** 2015-11-04

**Authors:** Chenchen Hu, Henghui Xu, Xiaoxiao Liu, Feng Zou, Long Qie, Yunhui Huang, Xianluo Hu

**Affiliations:** 1 State Key Laboratory of Materials Processing and Die & Mould Technology, School of Materials Science and Engineering, Huazhong University of Science and Technology, Wuhan 430074, P. R. China

## Abstract

VO_2_/TiO_2_ nanosponges with easily tailored nanoarchitectures and composition were synthesized by electrostatic spray deposition as binder-free electrodes for supercapacitors. Benefiting from the unique interconnected pore network of the VO_2_/TiO_2_ electrodes and the synergistic effect of high-capacity VO_2_ and stable TiO_2_, the as-formed binder-free VO_2_/TiO_2_ electrode exhibits a high capacity of 86.2 mF cm^−2^ (~548 F g^−1^) and satisfactory cyclability with 84.3% retention after 1000 cycles. This work offers an effective and facile strategy for fabricating additive-free composites as high-performance electrodes for supercapacitors.

Supercapacitors (SCs) are widely applied in portable electronics as well as smart-grid electrochemical energy storage, because of their higher energy densities compared to electrostatic capacitors and higher power densities compared to batteries^1^,^2^. According to the charge storage mechanism, there are two kinds of typical SCs: electric double layer capacitors (EDLCs) with charge absorbed on the surface of an electrode and pseudocapacitors (PCs) with reversible Faradic redox reactions on the surface and near-surface. Generally transition-metal oxides and conductive polymers are representative pseudocapacitive materials^3^. Their capacitance is generally 10 times larger than that of a carbon-based EDLC^4^. Among various transition-metal oxides, ruthenium oxides (RuO_*x*_) has been intensively investigated because of its attractive pseudocapacitive behaviour and stable long-term performance. However, the disadvantages of high cost and environmental harmfulness hamper its development in commercial applications. In this regard, several transition-metal oxides, such as manganese oxides^5^,^6^,^7^, vanadium oxides^8^,^9^,^10^,^11^, titanium oxides^12^,^13^,^14^ and their composites^15^,^16^,^17^,^18^ have been explored as the alternatives, due to their designed structures, excellent electrochemical performances and relatively reasonable price.

In practical applications, a high electrochemical performance and durability of a SC greatly depends on the combination of multiple factors, including the measurement techniques, electrode materials, electrolyte as well as the approach to assemble materials on the current collectors^19^. Normally in a typical fabrication process, a slurry of the mixture of binders, active materials and conductive additives with desired density is spread onto a proper current collector, followed by a drying and roll-pressing process to achieve a uniform coating layer^1^. However, the binders and other additives used in the fabrication process could induce several problems such as an increased resistivity and the addition of dead weight of an electrode^20^,^21^. Therefore, it is desirable to develop the binder-free and additive-free electrodes to overcome the mentioned problems.

To fabricate the binder-free electrodes, several techniques such as atomic layer deposition (ALD)^22^, vacuum filtration^23^,^24^, chemical vapor deposition (CVD)^25^,^26^,^27^, and electrospinning^4^,^28^,^29^ have been developed. Nevertheless, they always involve a disadvantage of expensive equipment, which hinders the large-scale production for practical applications^30^. In comparison, electrostatic spray deposition (ESD) is a versatile technique that has a cheap and simple set-up and can be worked under ambient conditions. It has been demonstrated successfully to fabricate various metal-oxide films with well-defined nano-structures^31^,^32^,^33^,^34^.

Herein, we fabricate binder-free VO_2_/TiO_2_ nanosponges by an ESD route and explore their electrochemical performance in SCs. The porous VO_2_/TiO_2_ film delivers a highest electrochemical capacitance of 86.2 mF cm^−2^ (~548 F g^−1^) between −1 and −0.3 V (*vs.* SCE) at a scan rate of 10 mV s^−1^. The three-dimensional (3D) pores grown on the porous nickel foam greatly enlarge the working area of film electrodes, help to facilitate the infiltration of electrolyte, thus largely enhance the electrochemical capacity^35^,^36^. Moreover, the rational design involving both high theoretical specific capacitance of vanadium oxides and high structural stability of titanium oxides improves the long-term durability during charge/discharge processes.

## Results and Discussion

**Figure 1:**
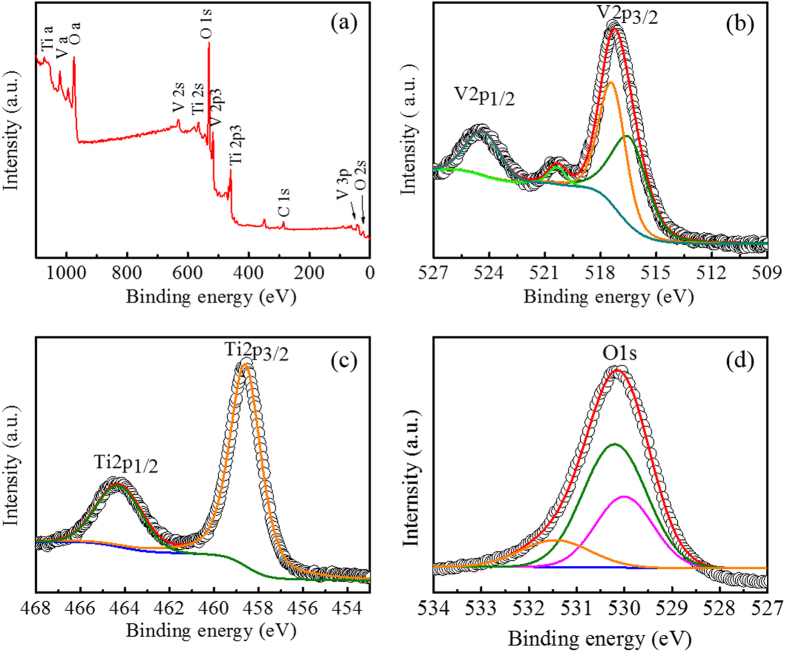
XPS spectra of the VT2 film. (**a**) Survey spectrum. High-resolution XPS spectra of (**b**) V 2p, (**c**) Ti 2p, and (**d**) O 1s.

For a detailed study, the films with an increasing molar ratio between V to Ti were prepared and marked as VT1, VT2 and VT3. To examine the surface electronic state and chemical composition, the products with different vanadium contents deposited on the glass slides followed by a certain heat treatment were characterized by X-ray photoelectron spectroscopy (XPS) (Fig. 1 and Fig. S1). A typical XPS spectrum for the VT2 film (Fig. 1a) indicates the existence of V, Ti, O and C elements. The high-resolution XPS spectrum for V2p_3/2_ can be deconvoluted into two peaks (Fig. 1c), where the peaks at 516.4 and 517.4 eV correspond to the reported binding energy for V^4+^ and V^5+^^10^,^37^. The existence of V^5+^ may result from the surface oxidation in air, which has been mentioned by other group^38^. The peaks at 464.28 and 458.60 eV are assigned to Ti2p_1/2_ and Ti2p_3/2_, which are characteristics of Ti(IV) in TiO_2_ (Fig. 1b)^39^,^40^. The O 1s in the XPS spectrum (Fig. 1d) can be fitted by three peaks centered at 530.0, 530.2 and 530.4 eV. The peak at 530.0 eV is ascribed to O 1s in the Ti-O linkage of TiO_2_, whereas the peak at 530.0 and 530.4 eV belong to the V-O linkage of VO_2_ and V_2_O_5_. In addition, the corresponding X-ray diffraction (XRD) patterns (Fig. S2) and energy-dispersive X-ray (EDX) (Fig. S3) analysis further confirm the phase and the composition of the films. Although VO_2_ and TiO_2_ can form a solid solution, the diffraction peaks in the XRD patterns of VT1, VT2 and VT3 films and powders indicate a mixture of monoclinic VO_2_ and anatase TiO_2_. This may due to the different formation steps of the two components. TiO_2_ is formed from the hydrolysis of TTIP during the early stage of deposition, while VO_2_ is formed after the certain heat treatment. However, Ti in TiO_2_ shares the same valence state and similar ion diameter to V in VO_2_, it is still very possible that a slight amount of Ti can be doped into VO_2_^41^. The atomic ratio between V to Ti from the EDX results present a good agreement with the composition in the precursor solution. This suggests that the composition of the products can be easily controlled and tailored as desired.

**Figure 2:**
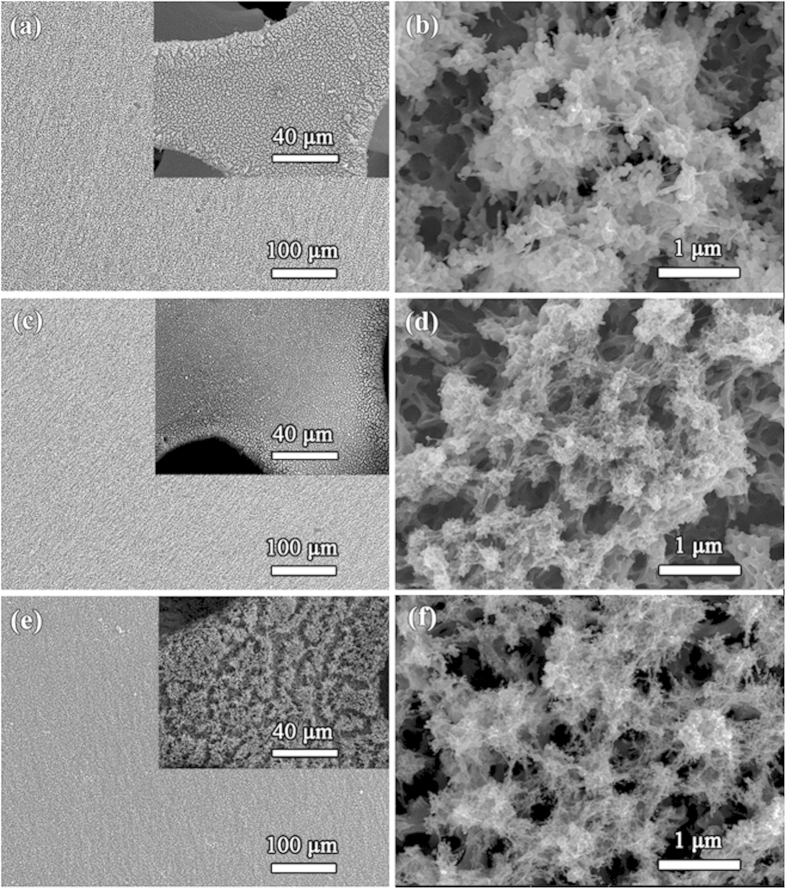
SEM images of the obtained thin films on the aluminum foil. (**a,b**) VT1, (**c,d**) VT2, and (**e,f**) VT3. The insets show the films on the nickel foam.

**Figure 3:**
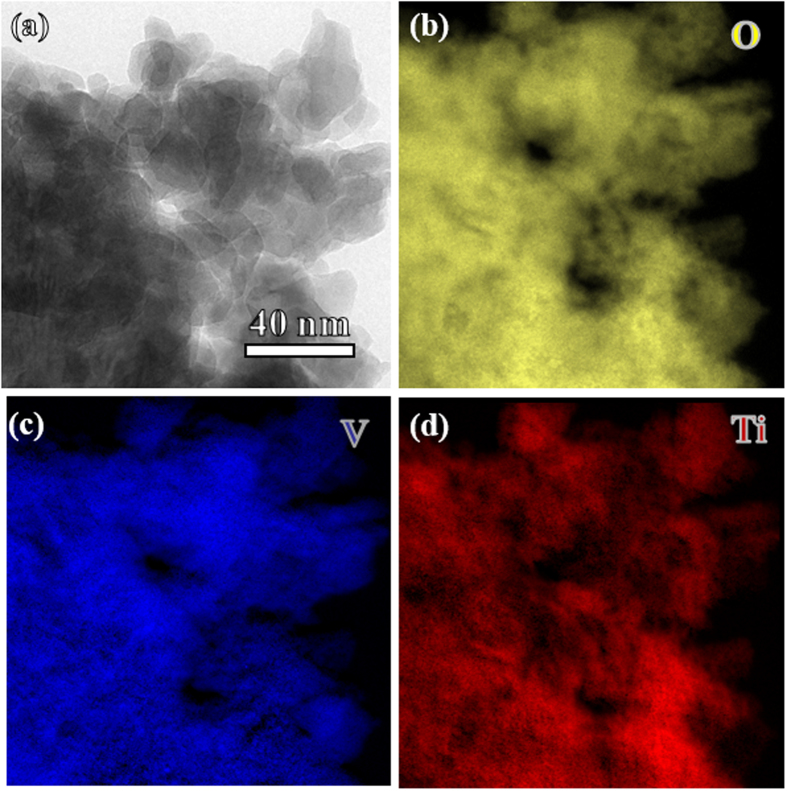
TEM morphologies of the VT2 film. (**a**) TEM image of VT2 film and elemental mapping images of (**b**) O, (**c**) V and (**d**) Ti.

The 3D porous morphologies of the as-formed films were revealed by field-emission scanning electron microscopy (FESEM) test. Figure 2a,c,e present the low-magnification FESEM images for the VT1, VT2, and VT3 films on the flat aluminum foil, while the insets present the films on the porous nickel foam. A highly uniformly distributed film on a flat aluminum at a large scale of 100 μm and even on a porous nickel foam (Fig. 2a,c,e) demonstrates a great advantage of the ESD technique – substrate-insensitive. The high-magnification SEM images (Fig. 2b,d,f) show that each film presents a 3D porous network structure with the diameter of the inner connected holes up to few micrometers. The skeleton of the porous sponge is comprised of tangled spherical particles and bunches, and their sizes depend greatly on the experimental conditions, including the flow rate, physical properties of the precursor solution and the applied voltage^42^. Generally, spray droplets become smaller with the increase of the applied voltage and decrease of the flow rate. This is also confirmed by the smallest particles in the VT3 film. The formation of such a highly porous structure can be ascribed to the two-step volatilization process caused by the solvent system^43^. The particles in VT3 show an average diameter less than 100 nm and the sticks are approximately 100 nm in length and 30 nm in diameter. Interestingly, the surface morphology remains almost unchanged during the heat treatment, indicating a highly stable feature of this 3D sponge. For comparison, the annealed powder from the dried precursor was also prepared. Results show that the shape of the resulting powder is irregular (Fig. S4). Scanning transmission electron microscopy (STEM) and X-ray elemental mappings characterization provide more insights into the detailed stucture. Figure 3a displays a representative TEM image for VT2 film. The diameter of the particles is around 20 to 40 nm, and some big holes between the particles could be beneficial to the infiltration of electrolytes. The STEM results (Fig. 3b,c,d) reveal that the TiO_2_ and VO_2_ nanoparticles are homogeneously distributed in nano-scale, indicating a stable combination of the two main components.

**Figure 4:**
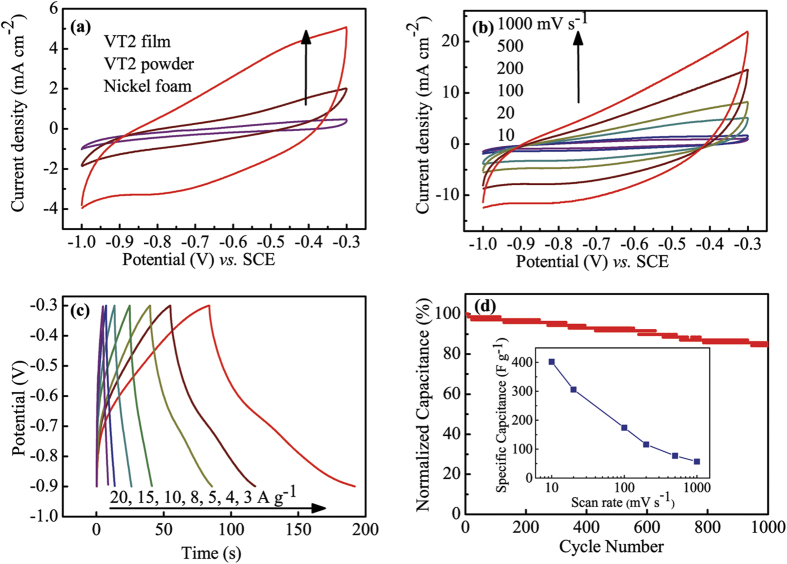
Electrochemical performance of the VT2 film. CV curves for (**a**) the VT2 film, the VT2 powder and the nickel foam at a scan rate of 100 mV s^–1^, and (**b**) the VT2 film at the scan rates of 10, 20, 100, 200, 500, and 1000 mV s^−1^. (**c**) Galvanoastatic charge-discharge curves of the VT2 film at current densities of 3, 4, 5, 8, 10, 15, and 20 A g^−1^. (**d**) Cycling performance at a current density of 10 A g^−1^ over 1000 cycles. The inset shows the specific capacitance as a function of scan rate ranging from 10 to 1000 mV s^−1^.

To evaluate the advantages of the 3D porous structure, the electrochemical performance of VT2 was measured in a three-electrode cell. An aqueous LiCl solution (8M) was selected as the electrolyte because of the negligible solubility and better cycling performance of vanadium oxides in it^22^,^44^. The thin films on the porous nickel foam and followed by an annealing treatment were used as the electrodes directly without adding any binders and other additives. The nickel foam performs as the current collector, ensuring a good pathway for electrons without introducing much extra capacitance (Fig. 4a). All the Cyclic voltammetry (CV) curves display a quasi-rectangular shape without obvious redox peaks, which may be caused by the low crystallinity of the particles and the selected potential window^9^. We also explored the electrode made of irregular powders with the traditional roll-pressing fabrication process. It is observed that the area enclosed by CV curves of the VT2 film triples that of powders at a scan rate of 100 mV s^−1^, implying a larger areal capacitance. Remarkably, the gravimetric capacitance of the electrodes made of the VT2 films calculated from the formula of *C*_m_ = *Q*/(2*Sρ*Δ*E*) is 6 times higher than that of the powders. This observation can be assigned to the unique 3D structure obtained by the ESD method. The interconnected pores provide higher specific area and more active sites for ion absorption and intercalation^45^. Figure 4b displays the CV curves of the VT2 film at various scan rates ranging from 10 to 1000 mV s^−1^. The maximum cathodic/anodic current density increases linearly with the scan rate, indicating a diffusion-controlled process. This can be attributed to the mass transport restrictions of the electrolyte, limiting the reaction with electrolyte species and active materials. According to the calculation using *C*_s_ = *Q*/(2*S*Δ*E*) and *C*_m_ = *Q*/(2*Sρ*Δ*E*), the thin film of VT2 exhibits a specific capacitance of 26.8 mF cm^−2^ (~174 F g^−1^) at the scan rate of 100 mV s^−1^, which agrees well with the previous results^17^,^18^. Figure 4c exhibits the galvanoastatic charge-discharge (GCD) curves at various current densities from 3 to 20 A g^−1^. The typical curves with a triangular shape show an obvious distortion, which is indicative of pseudocapacitive behaviour influenced by redox reactions along with electric double layer behaviour. A high specific capacitance of 715.8 F g^−1^ is obtained at a current density of 5 A g^−1^. It is observed that the *iR* drop increases with the increase of discharge current densities, but is still relatively small.

Furthermore, long-term cycling behaviour was investigated using the GCD measurement at a charge/discharge density of 10 A g^−1^ (Fig. 4d). After charging/discharging for 1000 cycles, the capacitance still remained 84.3% of the initial value. Notably, the Faradaic capacitance brings a large volumetric change in the vanadium oxide, thus leading to a severe decay of the electrochemical performance during the expansion/contraction process. However, the typical zero-strain material, anatase TiO_2_, can server as the stabilizer that restrains the volumetric change and keeps the framework stable during ion intercalation/deintercalation, therefore improving the cyclability. It is also reported that in the mixed system of titanium oxides and vanadium oxides, TiO_2_ prohibits a reorganization of the microstructure of the material, which improve the cycling stability^46^. Therefore, the largely improved electrochemical performance can be atributed to the 3D sponge-like structure, which increases the surface area and helps to improve the infiltration of the electrolyte as well as the accessibility of ions.

**Figure 5:**
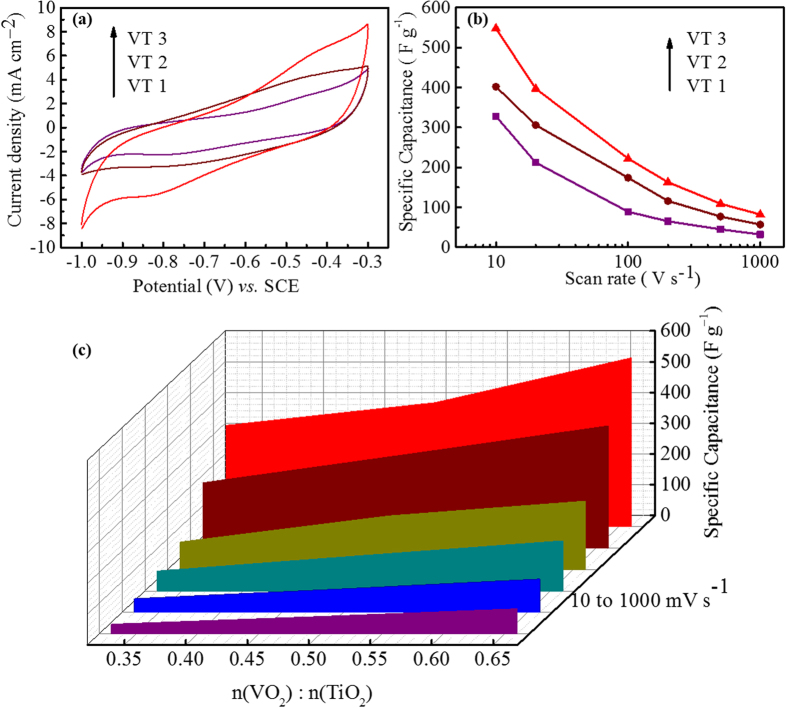
Electrochemical performances of VT1, VT2 and VT3 films. (**a**) CV curves at a scan rate of 100 mV s^−1^, (**b**) the specific capacitance as a function of scan rate and (**c**) the specific capacitance as a function of molar ratio between VO_2_ to TiO_2_ for the VT1, VT2 and VT3 films.

To further understand the influence of the VO_2_/TiO_2_ composition on the electrochemical performance, the electrodes with different molar ratios of VO_2_ to TiO_2_ were tested. Figure 5a exhibits the CV curves of the electrodes with different VO_2_/TiO_2_ ratios at a scan rate of 100 mV s^−1^. All the CV curves show symmetrical cathodic/anodic current peaks, implying a good reversibility of the electrodes made of 3D porous VO_2_/TiO_2_. Evidently, increasing the vanadium content can greatly enhance the current of redox reactions. The capacitances calculated from *C*_m_ = *Q*/(2*Sρ*Δ*E*) exhibit a linear increase to the molar percentage of VO_2_ (Fig. 5c), demonstrating a tailored capacitance through a designed compositions. Figure 5b summarized the rate capability calculated from CV measurements at scan rates between 10 to 1000 mV s^−1^. The thin film of VT3 exhibits the highest specific capacitance of 86.2 mF cm^−2^ (~548 F g^−1^) at the scan rate of 10 mV s^−1^. The specific capacitance can still be kept to be 13.0 mF cm^−2^ (~82 F g^−1^), when the scan rate reaches a high value of 1000 mV s^−1^. This is a satisfactory rate capability of pure metal oxide without any other additives. It can be ascribed to the decreased crystallite size as well as the increased surface area and the vanadium content in the VT3 film. The above results demonstrate that the additive-free thin-film electrodes of VO_2_/TiO_2_ exhibit an excellent supercapacitive performance. The interconnected pores provide an increased voids and defects, thus may lead to more active sites for redox reactions. Meanwhile, the monoclinic VO_2_ offers a high electrochemical capacity, while anatase TiO_2_ serving as a stabilizer both buffers the volumetric change and guarantees the long-term cyclability.

In summary, the binder-free VO_2_/TiO_2_ nanosponge electrodes have been successfully fabricated by an ESD technique. The electrodes present an easily tailored and controlled composition as well as the 3D sponge-like structure. Our results demonstrate that the additive-free VO_2_/TiO_2_ electrodes show a greatly enhanced electrochemical capacity of 86.2 mF cm^−2^ (~548 F g^−1^) at a scan rate of 10 mV s^−1^ and a satisfactory long-term stability of 84.3% retention at a current density of 10 A g^−1^. This superior supercapacitive performance can be ascribed to the unique highly porous structure that improves electrolyte infiltration and the zero-strain anatase TiO_2_. Furthermore, the facile and low-cost ESD route can be extended to fabricate other functional nanostructured thin films for energy-storage devices.

## Experimental Section

### Materials Synthesis

All the chemical reagents were commercially purchased and used directly without any purification. 0.0585 mg of NH_4_VO_3_ and 0.1576 mg of oxalic acid (C_2_H_2_O_4_) were dissolved into 10 ml of ethanol at 70 °C for 2 h, which is marked as solution A. 0.0716 mg of titanium (IV) isopropoxide (TTIP) was added into a mixture of 2.5 ml of ethanol and 2.5 ml of acetic acid, which is marked as solution B. A proper amount of solution A, i.e., 2.5, 5, and 10 ml, was added into solution B (5 ml) while vigorous stirring, which is marked as VT1, VT2, and VT3. Then 40 ml 1, 2-propylene was added into the solution.

The as-prepared precursor was transferred to a computer-controlled syringe pump, where the metallic needle was connected to a high-voltage power supply. Aluminum foils, nickel foams, and glass slides were chosen as the substrates for scanning electron microscopy (SEM), electrochemical measurements and X-ray diffraction (XRD) analysis, respectively. During the ESD procedure, the substrate was kept at 260 °C, and the distance between the substrate and the needle was 3 cm. The feeding rate and the applied voltage for VT1, VT2 and VT3 are shown in Table S1. To remove the excessive carbonaceous substances, the resulting thin films were thermally treated at 350 °C in air for 15 min before thermal treatment at 500 °C (heating rate : 1 °C min^−1^) under 5% NH_3_/Ar atmosphere for 90 min. For comparison, the precursor was dried at 260 °C and treated with the same heat treatment.

### Materials characterization

X-ray diffraction (XRD) analysis was performed by X’Pertb PRO (PANalytical B.V., Holland) Diffractometer with high-intensity Cu K_*α*1_ irradiation (*λ* = 1.5406 Å). The morphology was characterized using a field-emission scanning electron microscopy (FESEM, FEI, Sirion 200) coupled with an energy-dispersive X-ray (EDX, Oxford Instrument) spectrometer and transmission electron microscopy (TEM, JEOL 2100F). X-ray photoelectron spectroscopy (XPS) was measured on a VG MultiLab 2000 system with a monochromatic Al Kα X-ray source (ThermoVG Scientific).

### Electrochemical measurements

All the electrochemical performances were carried out on a CHI 660D electrochemical workstation. The SC tests were performed in a three-electrode cell with 8 M LiCl aqueous solution as the electrolyte and a Pt foil and a saturated calomel electrode (SCE) as the counter and reference electrodes, respectively. The substrates deposited with active materials were cut into pieces with average working area of ~1.2 cm^2^ and used as working electrodes directly. For comparison, the dried powder was mixed with polytetrafluoroethene (PTFE) and super P in a weight ratio of 80:10:10. The mixture was pressed onto the nickel foam for measurements. Cyclic voltammetry (CV) was performed at scan rates of 10 to 1000 mV s^−1^ over the potential range from −1.0 to −0.3 V (*vs*. SCE). Galvanostatic charge-discharge (GCD) measurements were carried out at current densities from 3 to 20 A g^−1^ and potential window between −0.9 and −0.3 V (*vs*. SCE). The calculation of the integrated-average volumetric and gravimetric capacitance of each electrode was according to: *C*_s_ = *Q*/(2*S*Δ*E*) and *C*_m_ = *Q*/(2*Sρ*Δ*E*), where *C*_s_ (F cm^−2^) and *C*_m_ (F g^−1^) is specific capacitance, *Q* (C) is the integrated cathodic charge, *S* (cm^2^) is the working area of electrodes, *ρ* (g cm^–2^) is the mass of active material per unit area, and Δ*E* (V) is the width of potential window. Considering for the accuracy and reproducibility, all electrodes were tested in order: CV (scan rates increasing from 10 to 1000 mV s^−1^) was followed by GCD (current densities increasing from 3 to 20 A g^−1^).

## Additional Information

**How to cite this article**: Hu, C. *et al.* VO_2_/TiO_2_ Nanosponges as Binder-Free Electrodes for High-Performance Supercapacitors. *Sci. Rep.* **5**, 16012; doi: 10.1038/srep16012 (2015).

## Supplementary Material

Supplementary Information

## References

[b1] Wang G. P., Zhang L. & Zhang J. J. A review of electrode materials for electrochemical supercapacitors. Chem. Soc. Rev. 41, 797–828 (2012). 21779609 10.1039/c1cs15060j

[b2] Lu X. H., Yu M. H., Wang G. M., Tong Y. X. & Li Y. Flexible Solid-State Supercapacitors: Design, Fabrication and Applications. Energy Environ. Sci. 7, 2160–2181 (2014).

[b3] Simon P. & Gogotsi Y. Materials for electrochemical capacitors. Nat. Mater. 7, 845–854 (2008). 18956000 10.1038/nmat2297

[b4] Chen X., Zhao B., Cai Y., Tade M. O. & Shao Z. Amorphous V-O-C composite nanofibers electrospun from solution precursors as binder- and conductive additive-free electrodes for supercapacitors with outstanding performance. Nanoscale 5, 12589–12597 (2013). 24177752 10.1039/c3nr04484j

[b5] Lee H. Y. & Goodenough J. B. Supercapacitor behavior with KCl electrolyte. J. Solid State Chem. 144, 220–223 (1999).

[b6] Yu Z. N., Duong B., Abbitt D. & Thomas J. Highly ordered MnO_2_ nanopillars for enhanced supercapacitor performance. Adv. Mater. 25, 3302–3306 (2013). 23636961 10.1002/adma.201300572

[b7] Xu H. *et al.* Flexible fiber-shaped supercapacitors based on hierarchically nanostructured composite electrodes. Nano Res. 8, 1148–1158 (2015).

[b8] Lee H. Y. & Goodenough J. B. Ideal Supercapacitor Behavior of Amorphous V_2_O_5_·nH_2_O in Potassium Chloride (KCl) Aqueous Solution. J. Solid State Chem. 148, 81–84 (1999).

[b9] Qu Q. T., Zhu Y. S., Gao X. W. & Wu Y. P. Core-Shell Structure of Polypyrrole Grown on V_2_O_5_ Nanoribbon as High Performance Anode Material for Supercapacitors. Adv. Energy Mater. 2, 950–955 (2012).

[b10] Wang G. M. *et al.* LiCl/PVA gel electrolyte stabilizes vanadium oxide nanowire electrodes for pseudocapacitors. ACS Nano 6, 10296–10302 (2012). 23050855 10.1021/nn304178b

[b11] Bao J. *et al.* All-solid-state flexible thin-film supercapacitors with high electrochemical performance based on a two-dimensional V_2_O_5_·H_2_O/graphene composite. J. Mater. Chem. A 2, 10876–10881 (2014).

[b12] Xia H. *et al.* Hierarchical TiO_2_-B nanowire@α-Fe_2_O_3_ nanothorn core-branch arrays as superior electrodes for lithium-ion microbatteries. Nano Res. 7, 1797–1808 (2014).

[b13] Lu X. H. *et al.* Hydrogenated TiO_2_ nanotube arrays for supercapacitors. Nano Lett. 12, 1690–1696 (2012). 22364294 10.1021/nl300173j

[b14] Wang Q., Wen Z. H. & Li J. H. A Hybrid Supercapacitor Fabricated with a Carbon Nanotube Cathode and a TiO_2_–B Nanowire Anode. Adv. Funct. Mater. 16, 2141–2146 (2006).

[b15] Dong S. M. *et al.* One dimensional MnO_2_/titanium nitride nanotube coaxial arrays for high performance electrochemical capacitive energy storage. Energy Environ. Sci. 4, 3502–3508 (2011).

[b16] Mai L. Q. *et al.* Cucumber-like V_2_O_5_/poly(3,4-ethylenedioxythiophene)&MnO_2_ nanowires with enhanced electrochemical cyclability. Nano Lett. 13, 740–745 (2013). 23311754 10.1021/nl304434v

[b17] Yang Y., Kim D., Yang M. & Schmuki P. Vertically aligned mixed V_2_O_5_-TiO_2_ nanotube arrays for supercapacitor applications. Chem. Commun. 47, 7746–7748 (2011). 10.1039/c1cc11811k 21647524

[b18] Yang Y., Kim D. & Schmuki P. Anodic formation of Ti-V binary oxide mesosponge layers for supercapacitor applications. Chem. Asian J. 6, 2916–2919 (2011). 21887749 10.1002/asia.201100488

[b19] Beidaghi M., Wang Z. F., Gu L. & Wang C. L. Electrostatic spray deposition of graphene nanoplatelets for high-power thin-film supercapacitor electrodes. J. Solid State Electrochem. 16, 3341–3348 (2012).

[b20] Presser V. *et al.* Flexible Nano-felts of Carbide-Derived Carbon with Ultra-high Power Handling Capability. Adv. Energy Mater. 1, 423–430 (2011).

[b21] Wang X. F. *et al.* Spray-Painted Binder-Free SnSe Electrodes for High-Performance Energy-Storage Devices. Chem Sus Chem 7, 308–313 (2014). 10.1002/cssc.201300241 24339208

[b22] Boukhalfa S., Evanoff K. & Yushin G. Atomic layer deposition of vanadium oxide on carbon nanotubes for high-power supercapacitor electrodes. Energy Environ. Sci. 5, 6872–6879 (2012).

[b23] Perera S. D. *et al.* Vanadium Oxide Nanowire-Carbon Nanotube Binder-Free Flexible Electrodes for Supercapacitors. Adv. Energy Mater. 1, 936–945 (2011).

[b24] Perera S. D. *et al.* Vanadium oxide nanowire – Graphene binder free nanocomposite paper electrodes for supercapacitors: A facile green approach. J. Power Sources 230, 130–137 (2013).

[b25] Warwick M. E. A., Roberts A. J., Slade R. C. T. & Binions R. Electric field assisted chemical vapour deposition – a new method for the preparation of highly porous supercapacitor electrodes. J. Mater. Chem. A 2, 6115 (2014).

[b26] Hao Sim D. *et al.* Direct growth of FeVO_4_ nanosheet arrays on stainless steel foil as high-performance binder-free Li ion battery anode. RSC Adv. 2, 3630 (2012).

[b27] Xia X. *et al.* A New Type of Porous Graphite Foams and Their Integrated Composites with Oxide/Polymer Core/Shell Nanowires for Supercapacitors: Structural Design, Fabrication, and Full Supercapacitor Demonstrations. Nano Lett. 14, 1651–1658 (2014). 24548206 10.1021/nl5001778

[b28] Kim B. H., Kim C. H., Yang K. S., Rahy A. & Yang D. J. Electrospun vanadium pentoxide/carbon nanofiber composites for supercapacitor electrodes. Electrochim. Acta 83, 335–340 (2012).

[b29] Mak W. F. *et al.* High-Energy Density Asymmetric Supercapacitor Based on Electrospun Vanadium Pentoxide and Polyaniline Nanofibers in Aqueous Electrolyte. J. Electrochem. Soc. 159, A1481–A1488 (2012).

[b30] Shakir I., Ali Z., Bae J., Park J.-J. & Kang D. J. Layer by Layer Assembly of Ultrathin V_2_O_5_ Anchored MWCNTs and Graphene on Textile Fabrics for High Energy Density Flexible Supercapacitor Electrodes. Nanoscale 6, 4125–4130 (2014). 24604248 10.1039/c3nr06820j

[b31] Nam K. W. & Kim K. B. Manganese oxide film electrodes prepared by electrostatic spray deposition for electrochemical capacitors. J. Electrochem. Soc. 153, A81–A88 (2006).

[b32] Kim I. H. & Kim K. B. Ruthenium oxide thin film electrodes prepared by electrostatic spray deposition and their charge storage mechanism. J. Electrochem. Soc. 151, E7–E13 (2004).

[b33] Wang S. Q., Li S. R., Sun Y., Feng X. Y. & Chen C. H. Three-dimensional porous V_2_O_5_ cathode with ultra high rate capability. Energy Environ. Sci. 4, 2854–2857 (2011).

[b34] Du J. & Choy K. L. Electrostatic spray assisted vapour deposition of TiO_2_-based films. Solid State Ionics 173, 119–124 (2004).

[b35] Ellis B. L., Knauth P. & Djenizian T. Three-Dimensional Self-Supported Metal Oxides for Advanced Energy Storage. Adv. Mater. 26, 3368–3397 (2014). 24700719 10.1002/adma.201306126

[b36] Xu J. *et al.* Three-Dimensional Structural Engineering for Energy-Storage Devices: From Microscope to Macroscope. Chem Electro Chem 1, 975–1002 (2014).

[b37] Zhai T. *et al.* A New Benchmark Capacitance for Supercapacitor Anodes by Mixed-Valence Sulfur-Doped V_6_O_13−x_. Adv. Mater. 26, 5869–5875 (2014). 25080307 10.1002/adma.201402041

[b38] Alov N., Kutsko D., Spirovová I. & Bastl Z. XPS study of vanadium surface oxidation by oxygen ion bombardment. Surf. Sci. 600, 1628–1631 (2006).

[b39] Qiao Y. *et al.* Conformal N-doped carbon on nanoporous TiO_2_ spheres as a high-performance anode material for lithium-ion batteries. J. Mater. Chem. A 1, 10375–10381 (2013).

[b40] Chen C. J. *et al.* Ionic-Liquid-Assisted Synthesis of Self-Assembled TiO_2_-B Nanosheets under Microwave Irradiation and Their Enhanced Lithium Storage Properties. Eur. J. Inorg. Chem. 2013, 5320–5328 (2013).

[b41] Takahashi K., Wang Y., Lee K. & Cao G. Fabrication and Li+-intercalation properties of V_2_O_5-_TiO_2_ composite nanorod arrays. Applied Physics A 82, 27–31 (2006).

[b42] Chen C. H., Kelder E. M., Jak M. J. G. & Schoonman J. Electrostatic spray deposition of thin layers of cathode materials for lithium battery Solid State Ionics 86–88, 1301–1306 (1996).

[b43] Yu Y., Chen C. H., Shui J. L. & Xie S. Nickel-foam-supported reticular CoO-Li_2_O composite anode materials for lithium ion batteries. Angew. Chem. Int. Ed. 44, 7085–7089 (2005). 10.1002/anie.200501905 16211641

[b44] Lao Z. J. *et al.* Synthesis of vanadium pentoxide powders with enhanced surface-area for electrochemical capacitors. J. Power Sources 162, 1451–1454 (2006).

[b45] Li H. Y. *et al.* Micelle anchored *in situ* synthesis of V_2_O_3_ nanoflakes@C composites for supercapacitors. J. Mater. Chem. A 2, 18806–18815 (2014).

[b46] Wang Y., Takahashi K., Lee K. H. & Cao G. Z. Nanostructured Vanadium Oxide Electrodes for Enhanced Lithium-Ion Intercalation. Adv. Funct. Mater. 16, 1133–1144 (2006).

