# Supplementary material for: VO2/TiO2 Nanosponges as Binder-Free Electrodes for High-Performance Supercapacitors
Source: Sci Rep. 2015 Nov 4;5:16012. doi: 10.1038/srep16012 (PMC4632158; doi:10.1038/srep16012)
Supplement: Supplementary Information [file srep16012-s1.doc]

**Supporting Information**

VO_2_/TiO_2_ Nanosponges as Binder-Free Electrodes for High-Performance Supercapacitors

Chenchen Hu, Henghui Xu, Xiaoxiao Liu, Feng Zou, Long Qie and Yunhui Huang & Xianluo Hu*

State Key Laboratory of Materials Processing and Die & Mould Technology, School of Materials Science and Engineering, Huazhong University of Science and Technology, Wuhan 430074, P. R. China.

Correspondence and requests for materials should be addressed to X.L.H. (huxl@mail.hust.edu.cn)

**Table S1** Experimental conditions and mass per area of annealed samples

| **Sample** | **Feed rate**  **(ml h^−1^)** | **Applied voltage**  **(kV)** | **Mass per area**  **(mg cm^−2^)** |
| --- | --- | --- | --- |
| VT1 | 0.9 | 14 | 0.1791 |
| VT2 | 1 | 16 | 0.1533 |
| VT3 | 0.7 | 16 | 0.1572 |

**Figure S1** High-resolution XPS spectrum of N 1s.

**Figure S2** XRD patterns for (a) annealed powders from the precursor only containing the Ti source, (b) annealed powders from the precursor only containing the V source, (c) the VT1 film and powder, (d) VT2 film and powder, and (e) VT3 film and powder.

**Figure S3.** TG/DTA result of porous hollow ZnO/ZnFe_2_O_4_/C octahedra obtained in the air flow at a heating rate of 10 °C min^−1^.


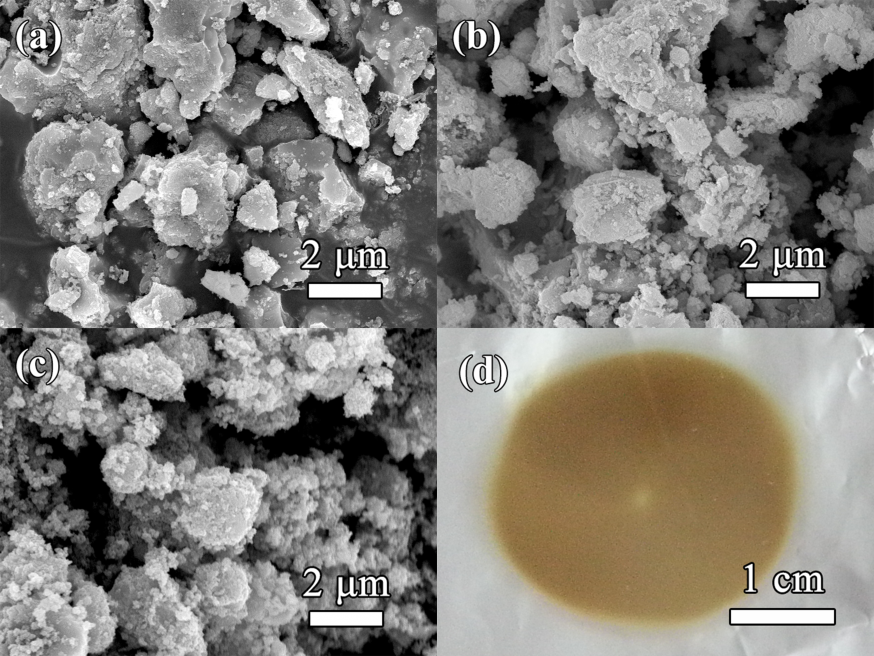


**Figure S4** SEM images of (a) VT1, (b) VT2, and (c) VT3 powders. (d) Digital photo of the as-prepared VO_2_/TiO_2_ film on an aluminum foil.
